# 2-Chloro-5-nitro­benzaldehyde thio­semicarbazone

**DOI:** 10.1107/S1600536810035701

**Published:** 2010-09-11

**Authors:** Yu-Mei Hao

**Affiliations:** a Department of Chemistry, Baicheng Normal University, Baicheng 137000, People’s Republic of China

## Abstract

The title Schiff base compound, C_8_H_7_ClN_4_O_2_S, was prepared by the reaction of equimolar quanti­ties of 2-chloro-5-nitro­benzaldehyde with thio­semicarbazide in methanol. The mol­ecule adopts a *trans* configuration with respect to the azomethine group and the dihedral angle between the benzene ring and the thio­semicarbazide group is 6.8 (3)°. In the crystal, mol­ecules are linked through inter­molecular N—H⋯S hydrogen bonds, forming chains propagating in [010].

## Related literature

For the crystal structures of similar Schiff base compounds, see: Ferrari *et al.* (1999 ▶); Shanmuga Sundara Raj *et al.* (2000 ▶); Chattopadhyay *et al.* (1988 ▶). For a similar compound reported by the author, see: Hao (2010 ▶). For reference structural data, see: Allen *et al.* (1987 ▶).
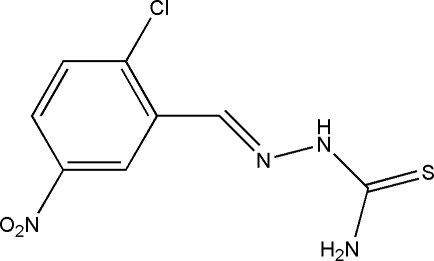


## Experimental

### 

#### Crystal data

C_8_H_7_ClN_4_O_2_S
*M* *_r_* = 258.69
Monoclinic, 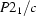

*a* = 11.611 (2) Å
*b* = 8.439 (2) Å
*c* = 12.016 (3) Å
β = 113.909 (2)°
*V* = 1076.4 (4) Å^3^
*Z* = 4
Mo *K*α radiation
μ = 0.54 mm^−1^
*T* = 298 K
0.18 × 0.17 × 0.17 mm

#### Data collection

Bruker SMART CCD diffractometer
Absorption correction: multi-scan (*SADABS*; Sheldrick, 1996 ▶) *T*_min_ = 0.909, *T*_max_ = 0.914
6657 measured reflections
2344 independent reflections
1573 reflections with *I* > 2σ(*I*)
*R*_int_ = 0.038

#### Refinement

*R*[*F* ^2^ > 2σ(*F* ^2^)] = 0.039
*wR*(*F* ^2^) = 0.111
*S* = 1.02
2344 reflections
154 parameters
4 restraints
H atoms treated by a mixture of independent and constrained refinement
Δρ_max_ = 0.20 e Å^−3^
Δρ_min_ = −0.17 e Å^−3^

### 

Data collection: *SMART* (Bruker, 2002 ▶); cell refinement: *SAINT* (Bruker, 2002 ▶); data reduction: *SAINT*; program(s) used to solve structure: *SHELXS97* (Sheldrick, 2008 ▶); program(s) used to refine structure: *SHELXL97* (Sheldrick, 2008 ▶); molecular graphics: *SHELXTL* (Sheldrick, 2008 ▶); software used to prepare material for publication: *SHELXL97*.

## Supplementary Material

Crystal structure: contains datablocks global, I. DOI: 10.1107/S1600536810035701/hb5633sup1.cif

Structure factors: contains datablocks I. DOI: 10.1107/S1600536810035701/hb5633Isup2.hkl

Additional supplementary materials:  crystallographic information; 3D view; checkCIF report

## Figures and Tables

**Table 1:** Hydrogen-bond geometry (Å, °)

*D*—H⋯*A*	*D*—H	H⋯*A*	*D*⋯*A*	*D*—H⋯*A*
N4—H4*A*⋯S1^i^	0.89 (1)	2.53 (1)	3.408 (2)	173 (2)
N3—H3⋯S1^ii^	0.90 (1)	2.46 (1)	3.3266 (19)	161 (2)

Symmetry codes: (i) 
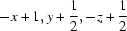
; (ii) 
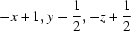
.

**Fig. 1.**
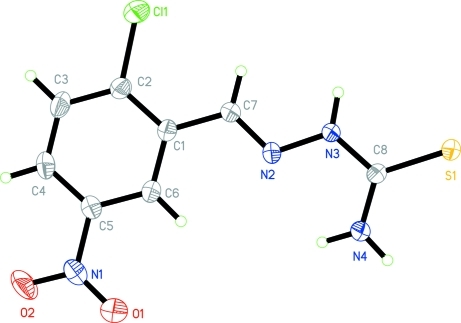
The molecular structure of the title compound with 30% probability ellipsoids.

**Fig. 2.**
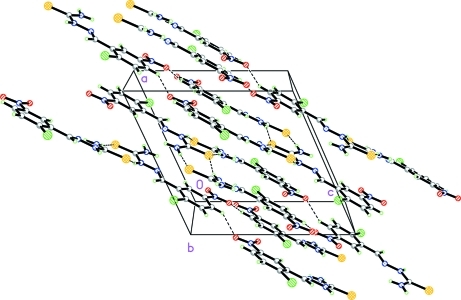
Crystal packing of the title compound with hydrogen bonds drawn as dashed lines.

## supplementary crystallographic information

### Comment

Recently, the crystal structures of a number of Schiff base compounds bearing the hydrazone groups derived from the thiosemicarbazide with aldehydes have been reported (Ferrari *et al.*, 1999; Shanmuga Sundara Raj *et al.*, 2000; Chattopadhyay *et al.*, 1988). Recently, the author has reported a Schiff base compound derived from the thiosemicarbazide with 2-hydroxy-4-methoxybenzaldehyde (Hao, 2010), in this paper, the title new Schiff base compound, (I), Fig. 1, is reported.

The molecule of the title compound adopts a *trans* configuration with respect to the azomethine group. All the bond lengths are within normal values (Allen *et al.*, 1987). The dihedral angle between the C1-C6 benzene ring and the plane defined by N2-N3-C8-S1-N4 is 6.8 (3)°, indicating the planar of the molecule. In the crystal structure, molecules are linked through intermolecular N—H···S hydrogen bonds (Table 1), to form chains (Fig. 2).

### Experimental

2-Chloro-5-nitrobenzaldehyde (0.1 mmol, 18.6 mg) and thiosemicarbazide (0.1 mmol, 9.1 mg) were refluxed in methanol (30 ml) for 30 min to give a clear yellow solution. Yellow blocks of (I) were formed by slow evaporation of the solvent over several days at room temperature.

### Refinement

H3, H4A and H4B were located from a difference Fourier map and refined isotropically, with the N—H and H···H distances restrained to 0.90 (1) Å and 1.43 (2) Å, respectively, and with *U*_iso_ restrained to 0.08Å^2^. Other H atoms were constrained to ideal geometries, with d(C—H) = 0.93 Å, and with *U*_iso_(H) = 1.2*U*_eq_(C).

### Crystal data

**Table:**

C_8_H_7_ClN_4_O_2_S	*F*(000) = 528
*M**_r_* = 258.69	*D*_x_ = 1.596 Mg m^−^^3^
Monoclinic, *P*2_1_/*c*	Mo *K*α radiation, λ = 0.71073 Å
Hall symbol: -P 2ybc	Cell parameters from 1613 reflections
*a* = 11.611 (2) Å	θ = 2.8–24.7°
*b* = 8.439 (2) Å	µ = 0.54 mm^−^^1^
*c* = 12.016 (3) Å	*T* = 298 K
β = 113.909 (2)°	Block, yellow
*V* = 1076.4 (4) Å^3^	0.18 × 0.17 × 0.17 mm
*Z* = 4	

### Data collection

**Table:**

Bruker SMART CCD diffractometer	2344 independent reflections
Radiation source: fine-focus sealed tube	1573 reflections with *I* > 2σ(*I*)
graphite	*R*_int_ = 0.038
ω scans	θ_max_ = 27.0°, θ_min_ = 3.0°
Absorption correction: multi-scan (*SADABS*; Sheldrick, 1996)	*h* = −14→14
*T*_min_ = 0.909, *T*_max_ = 0.914	*k* = −7→10
6657 measured reflections	*l* = −14→15

### Refinement

**Table:**

Refinement on *F*^2^	Primary atom site location: structure-invariant direct methods
Least-squares matrix: full	Secondary atom site location: difference Fourier map
*R*[*F*^2^ > 2σ(*F*^2^)] = 0.039	Hydrogen site location: inferred from neighbouring sites
*wR*(*F*^2^) = 0.111	H atoms treated by a mixture of independent and constrained refinement
*S* = 1.02	*w* = 1/[σ^2^(*F*_o_^2^) + (0.052*P*)^2^ + 0.1148*P*] where *P* = (*F*_o_^2^ + 2*F*_c_^2^)/3
2344 reflections	(Δ/σ)_max_ < 0.001
154 parameters	Δρ_max_ = 0.20 e Å^−^^3^
4 restraints	Δρ_min_ = −0.17 e Å^−^^3^

### Special details

**Table:**

Geometry. All esds (except the esd in the dihedral angle between two l.s. planes) are estimated using the full covariance matrix. The cell esds are taken into account individually in the estimation of esds in distances, angles and torsion angles; correlations between esds in cell parameters are only used when they are defined by crystal symmetry. An approximate (isotropic) treatment of cell esds is used for estimating esds involving l.s. planes.
Refinement. Refinement of F^2^ against ALL reflections. The weighted R-factor wR and goodness of fit S are based on F^2^, conventional R-factors R are based on F, with F set to zero for negative F^2^. The threshold expression of F^2^ > 2sigma(F^2^) is used only for calculating R-factors(gt) etc. and is not relevant to the choice of reflections for refinement. R-factors based on F^2^ are statistically about twice as large as those based on F, and R- factors based on ALL data will be even larger.

### Fractional atomic coordinates and isotropic or equivalent isotropic displacement parameters (Å^2^)

**Table:**

	*x*	*y*	*z*	*U*_iso_*/*U*_eq_	
Cl1	0.22502 (8)	−0.41154 (8)	0.48549 (7)	0.0669 (3)	
N1	0.11203 (19)	0.1863 (3)	0.67884 (19)	0.0541 (6)	
N2	0.33718 (17)	0.0503 (2)	0.40689 (17)	0.0402 (5)	
N3	0.39548 (18)	0.0489 (2)	0.32765 (18)	0.0409 (5)	
N4	0.4229 (2)	0.3150 (2)	0.3570 (2)	0.0543 (6)	
O1	0.13241 (19)	0.3120 (2)	0.64124 (19)	0.0694 (6)	
O2	0.0674 (2)	0.1755 (3)	0.7540 (2)	0.0965 (8)	
S1	0.50185 (6)	0.18861 (7)	0.19837 (6)	0.0460 (2)	
C1	0.2313 (2)	−0.0903 (3)	0.50752 (19)	0.0375 (5)	
C2	0.1926 (2)	−0.2336 (3)	0.5392 (2)	0.0424 (6)	
C3	0.1273 (2)	−0.2416 (3)	0.6133 (2)	0.0504 (6)	
H3A	0.1013	−0.3389	0.6314	0.061*	
C4	0.1014 (2)	−0.1031 (3)	0.6600 (2)	0.0506 (6)	
H4	0.0584	−0.1054	0.7107	0.061*	
C5	0.1405 (2)	0.0385 (3)	0.6300 (2)	0.0422 (6)	
C6	0.2036 (2)	0.0480 (3)	0.5549 (2)	0.0406 (5)	
H6	0.2275	0.1460	0.5360	0.049*	
C7	0.2974 (2)	−0.0829 (3)	0.4273 (2)	0.0407 (5)	
H7	0.3105	−0.1747	0.3912	0.049*	
C8	0.4371 (2)	0.1867 (2)	0.3007 (2)	0.0382 (5)	
H3	0.412 (2)	−0.0446 (19)	0.301 (2)	0.080*	
H4B	0.394 (3)	0.312 (3)	0.4146 (19)	0.080*	
H4A	0.450 (2)	0.4093 (18)	0.346 (2)	0.080*	

### Atomic displacement parameters (Å^2^)

**Table:**

	*U*^11^	*U*^22^	*U*^33^	*U*^12^	*U*^13^	*U*^23^
Cl1	0.1005 (6)	0.0371 (4)	0.0800 (5)	−0.0034 (3)	0.0541 (4)	0.0020 (3)
N1	0.0526 (13)	0.0621 (15)	0.0564 (14)	0.0075 (11)	0.0311 (11)	−0.0029 (11)
N2	0.0496 (11)	0.0372 (11)	0.0447 (12)	0.0008 (9)	0.0303 (9)	0.0032 (9)
N3	0.0569 (12)	0.0283 (10)	0.0533 (13)	0.0023 (9)	0.0385 (10)	0.0013 (9)
N4	0.0835 (16)	0.0338 (11)	0.0712 (15)	−0.0090 (11)	0.0578 (13)	−0.0081 (11)
O1	0.0868 (14)	0.0503 (12)	0.0874 (15)	0.0001 (10)	0.0521 (12)	−0.0058 (11)
O2	0.143 (2)	0.0869 (16)	0.1135 (18)	0.0285 (15)	0.1077 (17)	0.0100 (14)
S1	0.0628 (4)	0.0345 (3)	0.0580 (4)	−0.0034 (3)	0.0423 (3)	0.0004 (3)
C1	0.0400 (12)	0.0374 (13)	0.0381 (13)	−0.0023 (10)	0.0190 (11)	0.0026 (10)
C2	0.0497 (14)	0.0366 (12)	0.0432 (14)	−0.0023 (10)	0.0213 (11)	0.0017 (10)
C3	0.0563 (15)	0.0509 (15)	0.0503 (15)	−0.0118 (12)	0.0281 (13)	0.0073 (12)
C4	0.0515 (15)	0.0632 (17)	0.0483 (15)	−0.0046 (13)	0.0317 (12)	0.0042 (13)
C5	0.0416 (13)	0.0480 (14)	0.0413 (14)	0.0005 (11)	0.0214 (11)	−0.0010 (11)
C6	0.0431 (13)	0.0384 (13)	0.0457 (14)	−0.0031 (10)	0.0236 (11)	0.0037 (10)
C7	0.0496 (14)	0.0333 (12)	0.0486 (14)	0.0012 (10)	0.0294 (12)	−0.0009 (11)
C8	0.0435 (13)	0.0310 (12)	0.0460 (14)	−0.0001 (10)	0.0243 (11)	0.0023 (10)

### Geometric parameters (Å, °)

**Table:**

Cl1—C2	1.735 (2)	C1—C6	1.393 (3)
N1—O2	1.214 (3)	C1—C2	1.396 (3)
N1—O1	1.214 (3)	C1—C7	1.456 (3)
N1—C5	1.471 (3)	C2—C3	1.386 (3)
N2—C7	1.277 (3)	C3—C4	1.382 (4)
N2—N3	1.374 (2)	C3—H3A	0.9300
N3—C8	1.348 (3)	C4—C5	1.378 (3)
N3—H3	0.899 (10)	C4—H4	0.9300
N4—C8	1.322 (3)	C5—C6	1.376 (3)
N4—H4B	0.88 (3)	C6—H6	0.9300
N4—H4A	0.886 (10)	C7—H7	0.9300
S1—C8	1.681 (2)		
			
O2—N1—O1	123.3 (2)	C4—C3—H3A	120.5
O2—N1—C5	117.8 (2)	C2—C3—H3A	120.5
O1—N1—C5	118.9 (2)	C5—C4—C3	118.6 (2)
C7—N2—N3	116.36 (19)	C5—C4—H4	120.7
C8—N3—N2	118.95 (18)	C3—C4—H4	120.7
C8—N3—H3	121.7 (18)	C6—C5—C4	122.8 (2)
N2—N3—H3	119.1 (18)	C6—C5—N1	118.5 (2)
C8—N4—H4B	122.8 (17)	C4—C5—N1	118.6 (2)
C8—N4—H4A	122.1 (17)	C5—C6—C1	119.5 (2)
H4B—N4—H4A	115 (2)	C5—C6—H6	120.3
C6—C1—C2	117.5 (2)	C1—C6—H6	120.3
C6—C1—C7	120.4 (2)	N2—C7—C1	119.6 (2)
C2—C1—C7	122.1 (2)	N2—C7—H7	120.2
C3—C2—C1	122.6 (2)	C1—C7—H7	120.2
C3—C2—Cl1	117.04 (19)	N4—C8—N3	116.9 (2)
C1—C2—Cl1	120.40 (18)	N4—C8—S1	123.49 (17)
C4—C3—C2	119.1 (2)	N3—C8—S1	119.59 (16)

### Hydrogen-bond geometry (Å, °)

**Table:**

*D*—H···*A*	*D*—H	H···*A*	*D*···*A*	*D*—H···*A*
N4—H4A···S1^i^	0.89 (1)	2.53 (1)	3.408 (2)	173 (2)
N3—H3···S1^ii^	0.90 (1)	2.46 (1)	3.3266 (19)	161 (2)

Symmetry codes: (i) −*x*+1, *y*+1/2, −*z*+1/2; (ii) −*x*+1, *y*−1/2, −*z*+1/2.

## References

[bb1] Allen, F. H., Kennard, O., Watson, D. G., Brammer, L., Orpen, A. G. & Taylor, R. (1987). *J. Chem. Soc. Perkin Trans. 2*, pp. S1–19.

[bb2] Bruker (2002). *SAINT* and *SMART* Bruker AXS Inc., Madison, Wisconsin, USA.

[bb3] Chattopadhyay, D., Mazumdar, S. K., Banerjee, T., Ghosh, S. & Mak, T. C. W. (1988). *Acta Cryst.* C**44**, 1025–1028. 10.1107/s010827018800040x 3271092

[bb4] Ferrari, M. B., Capacchi, S., Pelosi, G., Reffo, G., Tarasconi, P., Albertini, R., Pinelli, S. & Lunghi, P. (1999). *Inorg. Chim. Acta*, **286**, 134–141. 10.1016/s0162-0134(00)00087-8 11001436

[bb5] Hao, Y.-M. (2010). *Acta Cryst.* E**66**, o2211. 10.1107/S1600536810029594 PMC3008052 21588582

[bb6] Shanmuga Sundara Raj, S., Fun, H.-K., Zhang, X.-J., Tian, Y.-P., Xie, F.-X. & Ma, J.-L. (2000). *Acta Cryst.* C**56**, 1238–1239. 10.1107/s0108270100010180 11025310

[bb7] Sheldrick, G. M. (1996). *SADABS* University of Göttingen, Germany.

[bb8] Sheldrick, G. M. (2008). *Acta Cryst.* A**64**, 112–122. 10.1107/S0108767307043930 18156677

